# The role of gender in the association between self-rated health and mortality among older adults in Santiago, Chile: A cohort study

**DOI:** 10.1371/journal.pone.0181317

**Published:** 2017-07-18

**Authors:** Ximena Moreno, Cecilia Albala, Lydia Lera, Hugo Sánchez, Alejandra Fuentes-García, Alan D. Dangour

**Affiliations:** 1 Institute of Nutrition and Food Technology, University of Chile.ElLíbano 5524, Macul, Santiago, Chile; 2 Public Health School, Faculty of Medicine, University of Chile. Av. Independencia 939, Santiago, Chile; 3 Department of Population Health, Faculty of Epidemiology and Population Health, London School of Hygiene & Tropical Medicine, London, United Kingdom; Mayo Clinic, UNITED STATES

## Abstract

**Background:**

Previous studies on the role of gender in the association between self-rated health and mortality have shown contrasting results. This study was aimed to determine the importance of gender in the association between self-rated health and mortality among older people in Santiago, Chile.

**Methods:**

A 10 year follow-up of 1066 people aged 60 or more, from the Chilean cohort of the Study of Health, Ageing and Well-Being. Self-rated health was assessed in face to face interviews through a single general question, along with socio-demographic and health status information. Cox proportional hazards and flexible parametric models for survival analyses were employed.

**Results:**

By the end of follow-up, 30.7% of women and 39.4% of men died. Adjusted hazard ratio of poor self-rated health, compared to good self-rated health, was 1.92(95% CI 1.29–2.86). In models stratified by gender, an increased risk of mortality was observed among women who rated their health as poor (HR = 2.21, 95% CI 1.43–3.40), but not among men (HR = 1.04, 95% CI 0.58–1.86). Age was associated with mortality in both groups; for men, functional limitation and underweight were also risk factors and obesity was a protective factor.

**Conclusions:**

Compared to older women who rated their health as good, older women who rated their health as poor had a 2 fold increased risk of mortality over the subsequent 10 years. These findings stress the importance of considering a gender perspective into health programmes, including those focused on older people, in order to address the different elements that increase, on the long run, the risk of dying among older women and men.

## Introduction

The relationship between self-rated health (SRH) and mortality among older people has been widely studied during the last three decades, and the existing evidence has shown a graded association between SRH and mortality among the older population [1–3]. However, there are some elements that indicate the need to provide new evidence on this research field.

First, several studies have found that the association disappeared after adjustment for other important covariates [4–8], and some studies have found less consistent results, depending on the SRH question used [9], follow-up length [10,11], age [12,13], and gender [14–17]. With respect to gender, results are diverging[18], suggesting greater effects among men [19, 20] or effects only among women [13,14,16,21], or among men [15,17,22–24].

Secondly, it is important to consider that many studies on the association between SRH and mortality among older adults do not include strategies to take into account time varying effects, although some examples exist [25–27]; furthermore, being Cox regressions the main analysis technique employed, the proportional hazards assumption is rarely verified. The previous should be considered in order to improve the accuracy of the risk estimation.

In addition, studies on the association between SRH and mortality among Latin American older adults are scarce [28–30], and have not to-date explored gender differences. It is important to find out if, as it has been observed in other regions of the world, a negative self-rating of health is significantly associated to mortality among older people. This has been studied mainly in developed countries with a slow ageing pattern, but the ageing process in the region is accelerated compared to European countries, and in contrast, living standards have not improved at a similar pace [31]. In the Chilean case, although the country has been ranked as a high income economy [32], and life expectancies in 2015 were among the highest of the region, with 85 years for women and 79 years for men [33], marked social inequalities persisted during the last decade, including avoidable differences related to income, in morbidity and mortality among the adult population [34,35], and among older adults [36,37], affecting more negatively healthy life expectancies of older women and the poorest.

Given the current sparse evidence, our study used a major Chilean cohort study to examine the association of SHR with mortality, with a particular focus on the importance of gender.

## Methods

This study was aimed to determine the importance of gender in the association between self-rated health and mortality among older people in Santiago, Chile. In order to do so, we considered a set of variables that could be associated to mortality and act as confounders or covariates in the studied association, including chronic diseases, functional limitation, smoking and body mass index, and socio-demographic variables that could modify this association.

The research was based on the Chilean sample of the Health, Well-being and Ageing (SABE) study, carried out in seven large cities of Latin America and the Caribbean. Methodological details of this study have been reported previously [38,39]. Briefly, the Chilean sample was obtained through a systematic recruitment of individuals aged 60 and over living in the community in Santiago in 1999–2000. Between October 1999 and March 2000, 1301 individuals (of 1563 eligible individuals) were contacted for a first general interview related to health and well-being. In the present study, we included 1066 of these individuals who were free of cognitive decline at the baseline interview, according to the Folstein and Pfeffer tests, validated for the Chilean population [40].

At the baseline assessment, participants underwent a structured interview in their homes that included questions on socio-demographic characteristics, prevalence of chronic diseases, smoking habits, and functionality. Anthropometric measurements were obtained by trained interviewers, including weight and height. SRH was determined by the question “How would you rate your overall health at the present time?” There were five response categories: excellent, very good, good, fair, and poor. Due to small numbers in the first two categories (1.9% and 4.2%, respectively), excellent, very good and good were collapsed and employed as the reference for analyses and compared with fair and good. To assess functional status, self-report of activities of daily living (ADL), instrumental activities of daily living (IADL) and physical performance or advanced activities of daily living (AADL) were obtained; functional limitation was determined according to the proposal developed by Albala et al. [41], as limitation in at least one ADL, or in two IADL, or in three AADL. To ascertain mortality, death certificates of the National Civil Registration Office were obtained up to end 2010.

The variables included in the analysis were number of self-reported chronic diseases (considering hypertension, diabetes, osteoarthritis, cancer, chronic obstructive pulmonary disease, myocardial infarction and stroke), functional limitation, smoking, body mass index, categorized according to the World Health Organization standard guidelines (<18.5 as underweight, 18.5–24.9 as normal, 25–29.9 as overweight, and 30 or more as obesity), age, gender and years of education.

Descriptive results are expressed as percentages. Kaplan Meier survival estimates were calculated for the three categories of SRH employed for the analyses. Cox proportional hazards regressions were carried out to estimate the risk of 10-year mortality; the dependent variable was survival time, measured in number of days from baseline until death or censorship for those still alive to Dec 31, 2010. The proportional hazards assumption was tested by adding an interaction term with time for each covariate, and time varying effects of body mass index was observed only in the model for men; in that case, a flexible parametric survival model was employed [42]. To build the models, the crude effect of each variable on mortality was assessed; every covariate with a significant effect was included in the adjusted model. A significant interaction between SRH and gender was identified; hence, models stratified by gender were carried out, employing the same strategy of covariates inclusion; smoking was not included in the final models. No interactions between SRH and age or between SRH and education were observed, Cox-Snell residuals were employed to test the goodness of fit of the models. All statistical analyses were performed using Stata 12 software (StataCorp, College Station, TX).

This study was approved by the ethics committees of the Pan-American Health Organization and the Institute of Nutrition and Food Technology of the University of Chile. All participants signed an informed consent to take part in the study.

## Results

The study sample had a mean age of 70.9 years (SD = 7.5) and contained almost twice as many women as men (Table 1). On average, men had more years of education, and women had more chronic diseases and were more likely to have functional limitation, to be widowed, and living alone. Men were more likely to report being current smokers. Women assessments of their own health tended to be more negative than those of men.

**Table 1:** Baseline characteristics of the sample.

	Total			Men		Women
Variables	(N = 1066)			(N = 358)		(N = 708)
*Mean (SD)* Age	70.9	(7.5)	70.3	(7.3)	71.2	(7.6)
*Mean (SD)* Education	5.6	(3.9)	6.4	(4.4)	5.2	(3.6)
Marital status						
*n(%)* Married	379	(35.6)	187	(52.2)	192	(27.2)
*n(%)* Single	74	(6.9)	11	(3.1)	63	(8.9)
*n(%)*Divorced	204	(19.1)	71	(19.8)	133	(18.7)
*n(%)*Widowed	409	(38.4)	89	(24.8)	320	(45.2)
*n(%)* Living alone	136	(12.8)	34	(10)	102	(14.4)
*n(%)* Smoking	127	(11.9)	61	(16.9)	66	(9.3)
*Mean (SD)* Chronicdisease	1.5	(1.2)	1.2	(1.1)	1.7	(1.2)
*n(%)*Functionallimitation	514	(48.3)	118	(33.4)	396	(55.9)
Self-rated health						
*n(%)*Good	397	(37.2)	158	(44.1)	239	(33.8)
*n(%)*Fair	454	(42.6)	140	(39.1)	314	(44.3)
*n(%)*Poor	215	(20.2)	60	(16.8)	155	(21.9)

By the end of follow-up period, which on average was 8.6 years (SD = 2.6 years), 141 men and 217 women had died, corresponding to 39.4% and 30.7% of them, respectively. Individuals who rated their health as fair or poor were more likely to die over the follow-up period, although this tendency was significant only among women (Table 2).

**Table 2:** Baseline self-rated health and vital status at the end of follow-up, by gender.

Self-rated health	Alive		Dead		
	n	%	N	%	p
Sample (N = 1066)					
Good	283	40	114	31.9	
Fair	306	43.2	149	41.6	<0.001
Poor	119	16.8	95	26.5	
Men (N = 358)					
Good	106	48.9	52	36.9	
Fair	78	35.9	63	44.7	0.083
Poor	33	15.2	26	18.4	
Women (N = 708)					
Good	177	36	62	28.6	
Fair	228	46.4	86	39.6	<0.001
Poor	86	17.5	69	31.8	

In the unadjusted model for the whole sample, the hazard ratio for fair SRH, compared to good, was 1.30 (95% CI 1.02–1.67), and the HR for poor SRH was 1.91 (95% CI 1.45–2.52); in fully adjusted models, including gender, age, education, chronic diseases, functional limitation and body mass index, the higher risk for fair SRH was not significant (HR 1.13 95% CI 0.80–1.61), and the HR for poor SRH was 1.92 (95% CI 1.29–2.56). There was a significant interaction term between SRH and gender (p = 0.013).

**Fig 1:**
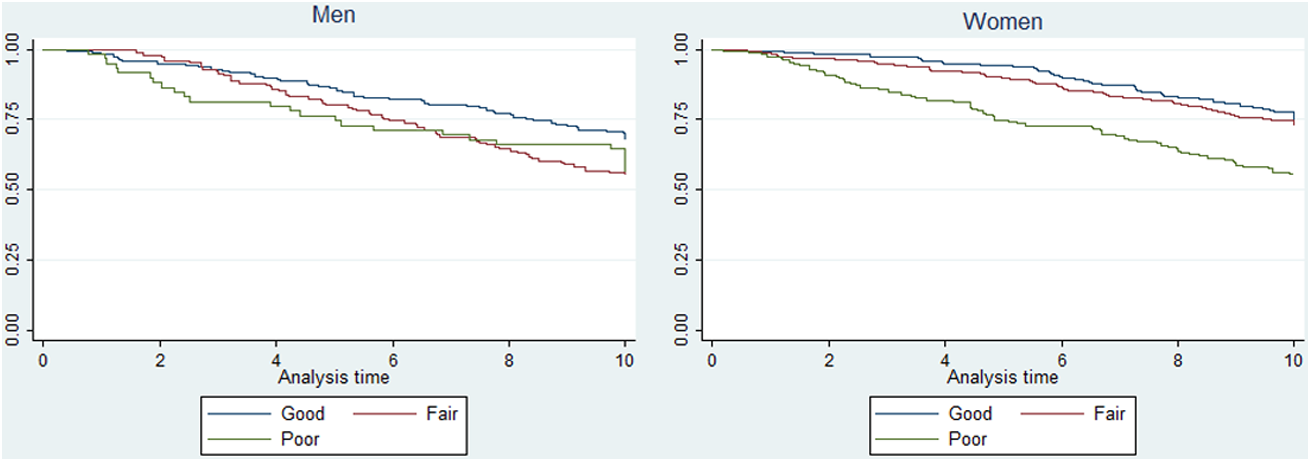
10 year survival estimates in men and women, according to baseline self-rated health.

The Kaplan Meier survival curves (Fig 1), suggest that women who rated their health as good or better had higher chances of survival, and the probabilities of survival decreased as the ratings of their health were more negative. In comparison, among men, the survival curves overlapped considerably during the observation period. Crude hazard ratios suggested a higher risk of dying among women who rated their health as poor, and among men, fair rating of health was associated with a higher risk of dying (Table 3).

**Table 3:** 10-year mortality among men and women in unadjusted and adjusted models.

Independent variable (Reference)	Category	Unadjusted model	Adjusted model
Women^a^			
Self-rated health (Good)	Fair	1.23 (0.88–1.72)	1.23 (0.86–1.78)
	Poor	2.49 (1.74–3.54)***	2.21 (1.43–3.40)***
Age (continuous variable)			1.09 (1.07-1-11)***
Education (0 years)			0.96 (0.93–1.00)
Chronic disease (None)			1.04 (0.91–1.20)
Functional limitation (No)			1.07 (0.76–1.50)
Body mass index (18.5–24.9)	<18.5		2.13 (0.96–4.72)
	25–29.9		0.80 (0.56–1.15)
	≥30		0.93 (0.64–1.34)
Men^b^			
Self-rated health (Good)	Fair	1.63 (1.12–2.37)**	1.17 (0.78–1.75)
	Poor	1.36 (0.84–2.21)	1.04 (0.58–1.86)
Age (continuos variable)			1.06 (1.04–1.09)***
Education (0 years)			1.01 (0.98–1.06)
Chronic disease (None)			1.15 (0.95–1.39)
Functional limitation (No)			1.66 (1.11–2.46)**
Body mass index^c^ (18.5–24.9)	<18.5		4.4 (1.29–14.96)*
	25–29.9		0.89 (0.60–1.34)
	≥30		0.58 (0.34–0.99)*

*p≤.05

** *p*≤.01

*** *p*≤.0001

a: Cox proportional hazards models

b: Flexible parametric survival model

c: Time varying effect.

In fully adjusted analyses, SRH was not associated with mortality among men; the variables that were significantly associated with an increased risk of mortality were age (HR = 1.06 95% CI 1.04–1.09) and functional limitation (HR = 1.66 95% CI 1.11–2.46); the same occurred with the category “underweight” (HR = 4.4 95% CI 1.29–14.96), whereas “obesity” was associated to a lower risk of dying by the end of follow-up (HR = 0.58 95% CI 0.34–0.99). Poor SRH was associated with higher mortality among women (HR = 2.21 95% CI1.43–3.40); the only other variable that showed a significant association with mortality was age (1.09 95% CI 1.07-1-11).

## Discussion

Poor SRH was associated with higher mortality among Chilean older adults, but the analysis stratified by gender showed that after controlling for age and objective health status, this association persisted only among women. These results confirm the importance of gender in the association between self-rated health and mortality.

Women who rated their health as poor had a higher risk of dying, and this was not explained by their worse baseline health status, as the adjusted model showed. This suggests that a worse baseline health status, characterised by multiborbidity and functional limitation, had a worse survival prognosis among women who rated their health as poor. This subjective negative assessment of health status could express, as Benyamini [1] proposes, an assessment of internal and external resources to cope with health problems, such as willingness to search for health care or to adhere to treatments, spending power, and the availability of family and social networks, all factors that could have an impact on future health trajectories and mortality.

Our results differed from what Idler [43] sets out, in order to explain the association between SRH and mortality, taking into account gender differences; according to this author, a higher proportion of negative self ratings of health among women are related to a higher prevalence of disability and chronic conditions, and when taking into account health status, the association between negative SRH and mortality disappears. In our study, the higher rates of disability and co-morbidities among women did not explain the association between poor self ratings of health and higher mortality; on the contrary, in the adjusted model, negative SRH was significantly associated to higher mortality risk, but not objective health status indicators. On the other hand, if men would have rated their health taking into account conditions that might have increased their mortality risk, whereas women would have tended to focus on disabling, but non fatal conditions, as Idler [43] proposes, an association between SRH and mortality among men would have been expected, or at least the association among women would not have been observed.

Our results showed that functional limitation and underweight were associated to a higher risk of mortality among men. Previously, Deeg [44] found that men show a worse trajectory related to functional limitation, with a higher risk of dying and a lower probability of recovery compared to women. Underweight in older people has also been described as a risk factor for diverse negative health outcomes, including mortality [45]. Both factors correspond to indicators of frailty, which relates to multiple negative health outcomes, including mortality [46]; the association between SRH and frailty and its impact on mortality is a research area that should be further explored; some existing evidence suggests that negative SRH does not increase the risk of dying among frail older people, but among those who are not frail [47]. The protective role of obesity that we found has been suggested by previous studies [48–50]; however, it is important to consider that BMI is not able to discern age related fat redistribution[51], and other measures could better perform as indicators of obesity, such as the waist hip ratio, which has shown to predict mortality in older people [52].

The results of our study have some clinical implications. First, SRH is a useful tool to identify older women at higher risk of mortality; it would be important to take into account this indicator as an input in the context of existing health programmes focused on preventing the progression of chronic diseases and the loss of functionality, in order to identify and cover the specific needs of women who rate their health as poor, and who could experience difficulties to search for health care and to adhere to treatments, including economic constrains and lack of networks, as suggested by Benyamini[1]. Second, the same health programmes should develop specific actions to early detect and treat older men at higher risk of adverse outcomes, including screening for frailty indicators such as functional decline and weight loss or underweight, and nutrition and exercise interventions aimed at recovery or delay of further decline.

One limitation of our study that should be considered is the fact that chronic diseases were collected by self-report. It is possible that a group of people were not aware of certain diagnoses, even if they had received them, due to patient-practitioner communication problems. It cannot be ruled out, also, the possibility of underreporting; in fact, the evidence suggests that the reliability of self report varies according to several socio-demographic characteristics and type of disease [53]; it is difficult to determine the existence or magnitude of this sort of bias in our study, due to the lack of information available about the reliability of self-report of chronic conditions among older people in Latin America. Another limitation is that our study was developed with a sample of people living in the urban area, with better average living conditions and better perceptions of health and quality of life compared to rural areas, as was observed in the National Study of Dependence among Older Adults conducted in 2009 in Chile [54], which makes our results not extend to older adults living in rural areas.The strengths of our study are that we followed-up a representative sample from the capital city, where 39 per cent of the older adults of the country live, that the outcome was ascertained for every subject, that time varying effects were considered in the analyses, and that a number of covariates related to health status, which are important to determine the existence of an association between SRH and mortality, were included.

## Conclusions

This study has reinforced the importance of gender in the association between self rated health and mortality. In the case of Chilean older women, those who rated their health as poor had a 2 fold increased risk of mortality over the subsequent 10 years, compared to those who rated it as good.

Our results highlight the need to integrate a gender perspective on health programmes focused on older adults, considering the higher risk of dying of older women who assess their health as poor. SRH is a simple test that should be routinely employed in primary care, along with the development or improvement of strategies for early detection of health problems, monitoring and adherence to treatments, and the maximisation of community and social resources that could have an impact on particular health trajectories of older women and men.

## Abbreviations

SRH: Self-Rated Health
SABE: Study on Health, Well-being and Ageing
ADL: Activities of Daily Living
IADL: Instrumental Activities of Daily Living
AADL: Advanced Activities of Daily Living
